# Supplementary material for: Developing an Olive Biorefinery in Slovenia: Analysis of Phenolic Compounds Found in Olive Mill Pomace and Wastewater
Source: Molecules. 2020 Dec 22;26(1):7. doi: 10.3390/molecules26010007 (PMC7792767; doi:10.3390/molecules26010007)
Supplement: Supplementary file 1 [file molecules-26-00007-s001.pdf]

Supplementary material

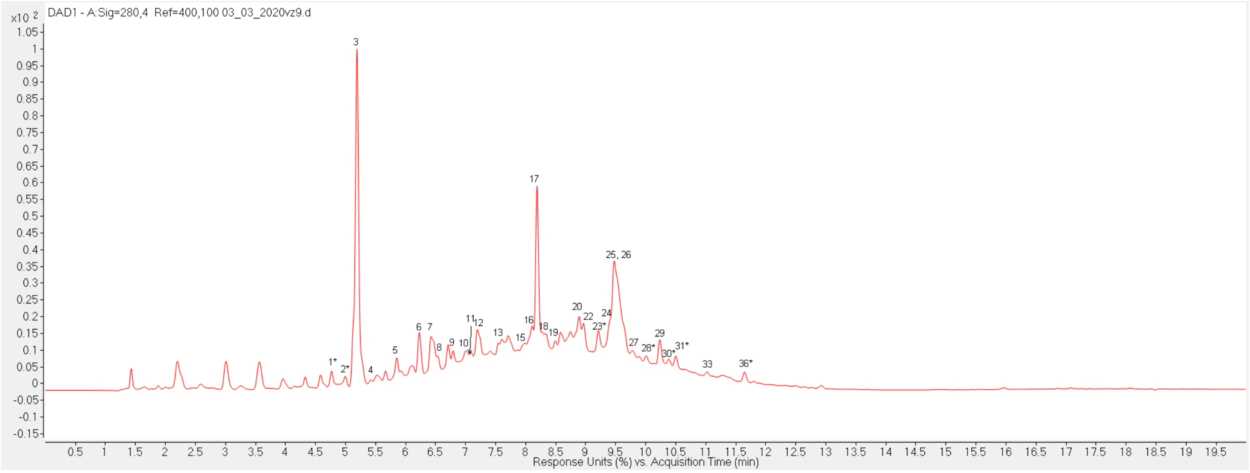

Supplementary Figure 1: An example of UV chromatogram at 280 nm of olive pomace extract.

Supplementary Table 1: Phenolic compounds found in pomace and in mill water.

Peak number	Compound	Fr.	RT (min) RRT*	Mr Exp.	Mr Calc.	Diff (ppm)	m/z [M]-	Fragments	Molecular formula	UV max (nm)
1	Oleoside**	P	4.8 0.92	390.1159	390.1162	-0.72	389.1089	389, 183, 209, 227	$C_{16}H_{22}O_{11}$	229, 289
2	Oleoside**	P	5.0 0.96	390.1163	390.1162	0.13	389.1091	389, 209, 345	$C_{16}H_{22}O_{11}$	255, 290
3	Hydroxytyrosol glucoside	P, W	5.2 1.00	316.1148	316.1158	-3.35	315.1071	315, 153, 123	$C_{14}H_{20}O_{8}$	230, 282
3	Hydroxytyrosol	P, W	5.2 1.00	154.0624	154.0630	-3.93	153.0551	123, 153	$C_{8}H_{10}O_{3}$	230, 280
3	Oleoside	P	5.2 1.00	390.1161	390.1162	-0.4	389.1090	389, 183, 209	$C_{16}H_{22}O_{11}$	200, 230, 280
4	Elenolic acid glucoside – Isomer 1	P	5.4 1.04	404.1321	404.1319	0.69	403.1244	403, 223, 179	$C_{17}H_{24}O_{11}$	236
4.1	Elenolic acid glucoside – Isomer 2	P	5.5 1.06	404.1320	404.1319	0.29	403.1248	403, 223, 179	$C_{17}H_{24}O_{11}$	235
5	Elenolic acid glucoside – Isomer 3	P	5.8 1.12	404.1317	404.1319	-0.43	403.1245	403, 223, 179	$C_{17}H_{24}O_{11}$	233

6	Tyrosol	P, W	6.2 1.19	/	/	/	/	/	$C_{10}H_8O_2$	227, 280
7	Secolagonoside	P, W	6.3 1.21	390.1160	390.3384	-0,49	389.1086	389, 345, 183, 209	$C_{16}H_{22}O_{11}$	230
8	Trans p-coumaric acid 4-glucoside	P	6.5 1.25	326.0994	326.1002	-2.49	325.0919	163, 119, 325	$C_{15}H_{18}O_8$	n.d.
9	Caffeic acid	P, W	6.7 1.29	180.0433	180.0423	5.55	179.0357	179, 135	$C_{16}H_{22}O_{11}$	230, 289, 330
10	Elenolic acid glucoside Isomer 4	P	7.0 1.35	404.1321	404.1319	0.67	403.1249	403, 223, 179	$C_{17}H_{24}O_{11}$	237
11	Luteolin-4`,7-*O*-diglucoside	P, W	7.1 1.37	610.1886	610.1898	-1.88	609.1795	609, 447, 285	$C_{27}H_{30}O_{16}$**	n.d.
12	β-OH-verbascoside Isomer I	P, W	7.2 1.38	640.2013	640.2003	1.45	639.1927	639, 621, 459, 179, 161	$C_{29}H_{36}O_{16}$	239 283 330
12	β-OH-verbascoside Isomer 2	P, W	7.2 1.38	640.2031	640.2003	4.27	639.1935	639, 621, 459, 179, 161	$C_{29}H_{36}O_{16}$	239 283 330
13	Vanilin	W	7.7 1.48	152.0477	152.0473	2.5	151.0406	151, 136	$C_8H_8O_3$	235 281 310
14	Verbascoside Isomer I	P	7.7 1.48	624.2087	624.2054	5.29	623.2018	623, 461, 161	$C_{29}H_{36}O_{15}$	265, 291, 330
15	Demethyloleuropein	P, W	7.9 1.52	526.1704	526.1686	3.33	525.1623*	525, 389, 319, 183, 345	$C_{24}H_{30}O_{13}$	240 280
16	Rutin	P, W	8.1 1.56	610.1557	610.1534	3.72	609.1469	609, 300, 179	$C_{27}H_{30}O_{16}$	256 358
17	Verbascoside Isomer II	P	8.2 1.58	624.2057	624.2054	0.47	623.1981	623, 461, 161	$C_{29}H_{36}O_{15}$	247 285 331
18	Luteolin-7`-*O*-glucoside	P, W	8.3 1.60	448.1014	448.1006	1.76	447.0938	447, 285	$C_{21}H_{20}O_{11}$	255 350
18	Luteolin rutinoside	P, W^x	8.3 1.60	594.1605	594.1585	3.47	593.1533	593, 285, 447	$C_{27}H_{30}O_{15}$	255 350

19	Nuzhenide Isomer 1	P	8.4 1.62	686.2392	686.2422	-4.4	685.2334	685, 523, 453, 421, 299, 223	$C_{31}H_{42}O_{17}$	239 277 333**
20	Luteolin-4`-*O*-glucoside	P, W	8.9 1.71	448.1010	448.1006	1.06	447.0934	447, 285	$C_{21}H_{20}O_{10}$	285, 330
21	Caffeoyl-6-secologanoside	P, W	8.9 1.71	552.1479	552.1479	0.02	551,1406	551, 507, 393, 281, 251, 179, 161	$C_{25}H_{28}O_{14}$	235, 325
22	Nuzhenide Isomer 2	P	9.0 1.73	686.2427	686.2422	0.68	685.2365	223, 299, 453, 523, 685	$C_{31}H_{42}O_{17}$	242 280, 330
23	Luteolin-3`-*O*-glucoside**	P, W	9.3 1.79	448.1018	448.1006	2.71	447.0939	447, 285	$C_{21}H_{20}O_{11}$	280
24	Oleuropein	P	9.4 1.81	540.1844	540.1843	0.26	539.1770	539, 149, 275, 377, 223	$C_{25}H_{32}O_{13}$	233, 282
25	3,4-DHPEA-EDA	P	9.5 1.83	320.1269	320.1260	2.77	319.1185	195, 183, 165, 139	$C_{17}H_{20}O_{6}$	237, 282
26	Oleuropein aglycone Isomer 1**	P	9.5 1.83	378.1320	378.1315	1.43	377.1245	377, 275, 149, 139, 307	$C_{19}H_{22}O_{8}$	n.d.
27	Oleuropein/Oleuroside	P	9.7 1.87	540.1822	540.1843	-3.92	539.1761	377. 539, 275, 149	$C_{25}H_{32}O_{13}$	239
28	Oleuropein aglycone Isomer 2**	P	10.0 1.92	378.1328	378.1315	3.44	377.1250	377, 345, 275, 149, 139, 307	$C_{19}H_{22}O_{8}$	225, 275
28	Oleuropein/Oleuroside **	P, W	10.0 1.92	540.1813	540.1843	-5.57	539.1743	275, 539, 149	$C_{25}H_{32}O_{13}$	225, 275
29	Ligstroside	P, W*	10.3 1.98	524.1889	524.1894	-0.82	523.1812	523, 223, 101	$C_{25}H_{32}O_{12}$	252, 270, 350
29.1	Oleuropein aglycone Isomer 3	P	10.3 1.98	378.1318	378.1315	0.78	377.1240	377, 345, 275, 149, 139, 307	$C_{19}H_{22}O_{8}$	240, 270
30	p-HPEA-EDA **	P	10.4 2.00	304.1312	304.1311	0.38	303.1235	179, 165, 183*, 59*, 137*	$C_{17}H_{20}O_{5}$	230, 282
30	Oleuropein aglycone Isomer 4 **	P	10.4 2.00	378.1321	378.1315	1.64	377.1234	377, 345, 275, 149, 139, 307	$C_{19}H_{22}O_{8}$	230, 280

31	Oleuropein aglycone Isomer 5 **	P	10.5 2.02	378.1314	378.1315	-0.12	377.1240	377, 345, 275, 149, 139, 307	$C_{19}H_{22}O_8$	225 280
32	Oleuropein aglycone Isomer 6	P	10.7 2.06	378.1327	378.1315	3.33	377.1242	377, 345, 275, 149, 139, 307	$C_{19}H_{22}O_8$	n.d.
33	Apigenin	P, W	11.0 2.12	270.0530	270.0523	0.71	269.0457	269	$C_{15}H_{10}O_5$	239, 269, 339
34	Oleuropein aglycone Isomer 7	P, W	11.1 2.13	378.1322	378.1315	2.02	377.1243	377, 275, 149, 139, 307, 327	$C_{19}H_{22}O_8$	n.d.
35	3,4-DHPEA-EDA	P	11.3 2.17	320.1262	320.1260	0.62	319.1187	195, 183, 165, 139	$C_{17}H_{20}O_6$	232, 280
35	Oleuropein aglycone Isomer 8 **	P	11.3 2.17	378.1319	378.1315	1.03	377.1242	377, 275, 149, 139, 307, 327	$C_{19}H_{22}O_8$	230 280
36	Oleuropein aglycone Isomer 9 **	P	11.6 2.23	378.1315	378.1315	0.06	377.1242	377, 275, 149, 139, 307, 327	$C_{19}H_{22}O_8$	225, 282

Relative retention time (RRT) was calculated with the respect of the retention time of the standard hydroxytyrosol.
